# New Endovascular Method for Transvascular Exit of Arteries and Veins: Developed in Simulator, in Rat and in Rabbit with Full Clinical Integration

**DOI:** 10.1371/journal.pone.0010449

**Published:** 2010-05-03

**Authors:** Johan Lundberg, Stefan Jonsson, Staffan Holmin

**Affiliations:** 1 Department of Clinical Neuroscience, Karolinska Institutet and Department of Neuroradiology, Karolinska University Hospital, Stockholm, Sweden; 2 Department of Materials Science and Engineering, Royal Institute of Technology, Stockholm, Sweden; Cuban Neuroscience Center, Cuba

## Abstract

**Background:**

Endovascular technique has benefits *vis-a-vis* surgical access to organs with less accessible anatomical locations. To minimize surgical risk we propose a novel endovascular technique, to create parenchymal access through endovascular methods.

**Methodology/Principal Findings:**

We have developed, manufactured and tested an endovascular catheter with a depth limiting collar and a penetrating tip that is used to perforate vessels, thereby creating a working channel to the extra-vascular space. Computer simulations and subsequent interventions have been performed *ex vivo* and *in vivo* in both small and large animals by testing different prototypes. All tests were designed for testing extravascular hemostasis and absence of thrombo-embolic complications when exiting the vessels from the inside to the extra vascular space. We have deposited prototypes after intervention in vascular walls over a period of 14 days in rat with no impairment on blood flow and no signs of thrombo-embolic complications upon re-exploration (n = 7). We have also incorporated the catheter system with clinically available systems both in an *ex vivo* simulator setting and in a full scale clinical angiographical setting in rabbit were no bleeding (0%) in any of the interventions performed (n = 40). To prevent hemorrhage during termination of the procedure, a hollow electrolysis detachment-zone leaves the distal tip in the vessel-wall after the intervention. This has also been tested with absolute hemostasis in large animals (n = 6).

**Conclusions/Significance:**

We have developed and tested a new system for transvascular tissue access in simulations, *ex vivo* and *in vivo* in small and large animals, integrating it with standard clinical catheters and angiographical environment, with absolute hemostasis and without thromboembolic complications. In a clinical setting for stem cell transplantation, local substance administration or tissue sampling, the benefit should be greatest in organs that are difficult or high-risk to access with other techniques, such as the pancreas, the central nervous system (CNS) and the heart.

## Introduction

There is a trend towards minimally invasive techniques for transplantation of stem cells, local administration of substances or sampling from various organ systems. Most organs and tissues in the body can be reached by needles with or without ultrasonic or computerized tomography guidance. If that is not possible, open surgery is an option as is stereotactic delivery assisted by modern imaging techniques [1]. For organs with less accessible anatomical location, parenchymal access can be associated with significant surgical risks [2]. The development of endovascular micro-catheter techniques has opened a possibility to reach virtually all parts of the body via the “internal routes” that arteries and veins constitute. This has led to the possibility to perform minimally invasive transplantations [3], [4].

Advancements in stem cell research have created a potential for regenerative treatment strategy to a wide spectrum of diseases tested both in experimental animal systems and in humans, *e.g*. diabetes mellitus [5], Parkinson [6], ischemic heart disease [7], traumatic brain injury [8] and stroke [9]. Preferably, minimally invasive techniques for reaching a desired target organ should be used. In one comparison of different techniques for administration to the central nervous system following stroke, the most efficient way of transplantation was intra-cerebral followed by intra-cerebroventricular and then intravenous delivery [10]. In a previous study we have also shown that the intra-arterial route gives a higher engraftment ratio *vis-à-vis* the intravenous route in a rat traumatic brain injury model [11]. To accomplish successful transplantation of stem cells, a few considerations must be made, *e.g.* accessibility to target organ, cell-type, volume and success-rate in engraftment [3]. It is probable that some cells possess a homing feature mediated through receptor-ligand interactions [12]. For cells with those properties an intravenous route would possible be favorable giving better distribution throughout the transplantation target zone [10]. In situations where the cellular engraftment rate after endovascular administration is low and when a high anatomical specificity for the engraftment is required, direct puncture of the parenchyma is preferable. This can be done with guided percutaneous needle puncture or in a combination with open surgery [13]. The transvenous system described by Thompson et al. [14] adds a possibility to, via large veins, administrate cells transvenously to the heart. The design of their device with a large diameter catheter and without a closure device for the penetration site, makes it usable predominantly in large vessels on the venous side, more specifically in the coronary sinus of the heart [14], [15]. The pancreas is however not reachable but it has been suggested that it would be of great benefit if insulin producing cells could be transplanted directly to the parenchyma [16].

We now propose a new method using an endo-luminal device herein named Extroducer, for exiting the micro- or macrovasculature throughout the body on both the arterial and the venous side. The design and concept of the device is associated with Seldingers original work describing the introducer [17]. A standard endovascular clinical catheter system, including an introducer, a guidecatheter and a microcatheter, is navigated within the vasculature to any target organ. Once the microcatheter is in the desired location within the microvasculature, the Extroducer system is advanced through the microcatheter. The Extroducer then safely penetrates the arterial or venous wall, as a nanocatheter, to reach the extravascular space, e.g. the parenchyma of any desired organ. The system thereby creates a working channel in order to be able to administrate or sample cells and substances to/from the extra-vasal space, and to make closure of the vessel wall safe. The rationale behind this technique is to combine minimal invasiveness of an endo-luminal approach with an accurate administration in a desired anatomical location. The exact relation between a specified vessel segment and a desired parenchymal target zone is today easily recognized with modern imaging techniques.

In previous works with intravascular transplantation of cells, there is little discussion about the control over cellular engraftment location, or the somewhat unfavorable ratio between transplanted and engrafted cells. The aim of this study and the development of the Extroducer concept are to combine favorable properties of minimal invasiveness with accurate and efficient engraftment of stem cells, and additionally, to make local administration of any substance, such as cytostatics, isotopes, growth factors, contrast agents, puncture of cysts or sampling in difficult anatomical locations possible.

## Results

### Extroducer concept

Interventions have been performed *ex vivo* and *in vivo* in both small and large animals by testing Extroducer prototypes. All tests demonstrate absolute extravascular hemostasis and absence of thrombo-embolic complications when exiting the vessels from the inside to the extra vascular space. We have deposited prototypes after intervention in vascular walls over a period of 14 days in small animals with no impairment on blood flow or signs of thrombo-embolic complications upon re-exploration. We have also incorporated the Extroducer system with clinically available catheter-systems both in an *ex vivo* simulator setting and in a full scale clinical angiographical setting in large animals.

We first tested the concept of performing an exit through an arterial wall in the tail artery of the rat with a sharply cut nitinol tube acting as both introducer and Extroducer. This showed that no hemorrhage occurred in either penetration from outside and in and vice versa from inside and out providing rationale for expanding the study.

### Stop design, hollow detachment zone and effects on cells

**Figure 1:**
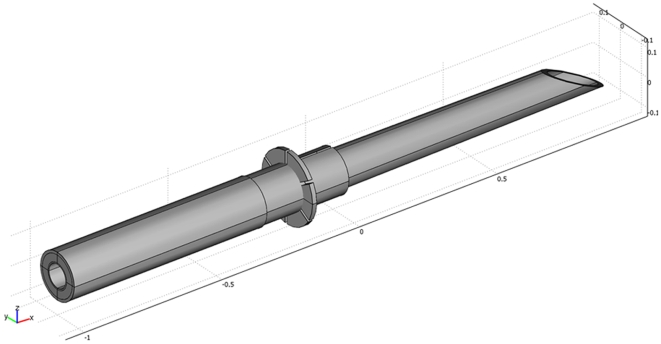
Principal design of the tip of the Extroducer. Different designs of intrusion depth limiting collars were tested for optimal stopping ability in a safe manner. Here, a principal drawing of the design is depicted with an intrusion depth limiting collar.

Conceptual prototypes (fig. 1) formed the basis for further testing by solving two problems; a depth limiting device for exact control of the system in a clinical angiographical setting and a detachment zone, thereby leaving the distal part as a plug in the vessel wall to prevent hemorrhage and thus providing a closure method after completion of the intervention. Prototypes were all constructed by hand and electrolysis detachments in isotonic saline solution were successful at 8 V in an average time of 20 seconds.

We tested mesenchymal stem cells in a nitinol system with the smallest lumen diameter available (ID 0.104±0.0127 mm) with 1700 mm length by counting viable/dead cells after passage. This showed that 10% died, but the remaining 90% of the cells survived and were possible to re-seed *in vitro*. We have also showed effect of passage on stem cell cultures in a previous published paper, albeit in a shorter nitinol tube[11].

**Figure 2:**
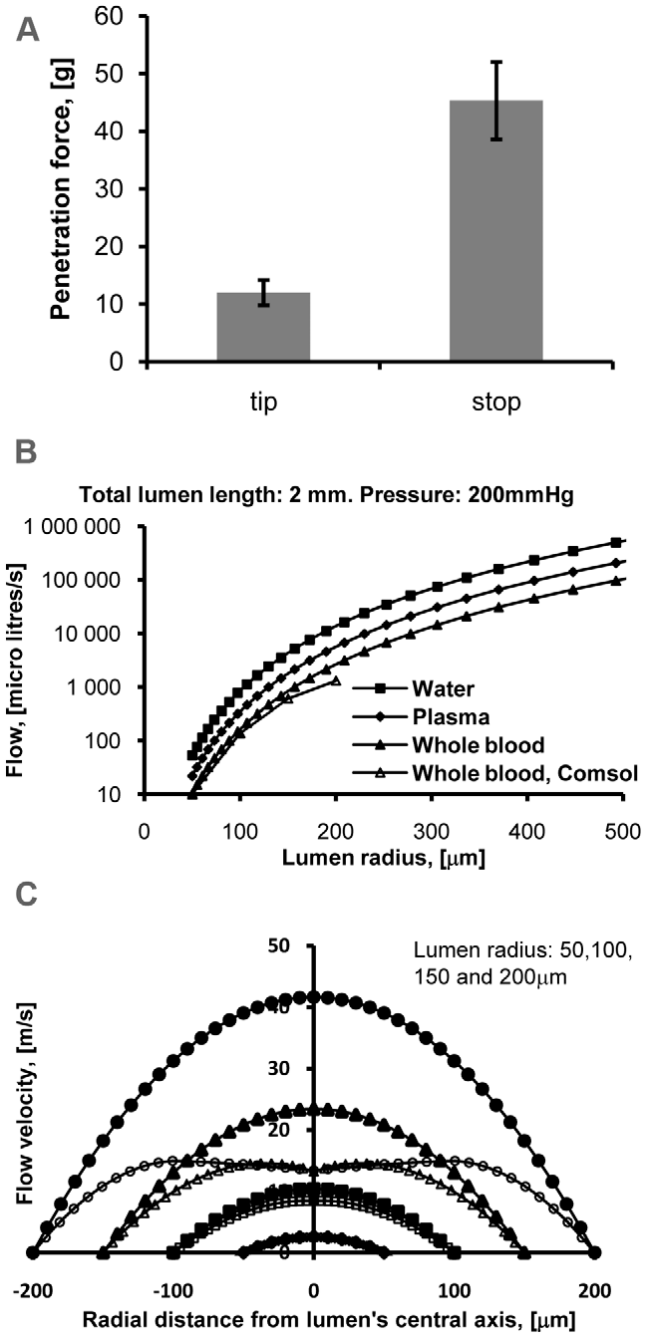
*Ex vivo* and simulation data from penetration forces and blood-flow in the catheter lumen. In a. graph showing data from a loading cell connected to a longitudinally cut aorta mounted in free air with the first bar corresponding to force applied to penetrate the vessel wall from inside and out and the second bar corresponding to perforation by the depth limiting collar, an “overshooting” of the system. Error bars are Standard deviations. In b. graph showing flow rates (Y-axis) plotted against lumen radius (X-axis). The flow rate becomes small as the lumen radius is reduced. At radii over 50 micrometer, turbulent flow gives lower flow rates (open symbols) which is taken into account in the calculations with COMSOL Multiphysics as compared to a perfect laminar flow (filled symbols). In c. graph illustrating velocity fields of circular Poiseuille flows (filled symbols) and COMSOL Multiphysics (open symbols) in an Extroducer device with a 2 mm long lumen, wherein the velocity fields, driven by a pressure of 200 mmHg (Y-axis), are plotted against different lumen radius (X-axis). This shows turbulence impact in reducing the velocity in the central part of the velocity fields. At a 50 micrometer radius COMSOL Multiphysics is identical to the circular Poiseuille flow but at higher radii the impact of turbulence becomes apparent.

We then performed *ex vivo* tests on longitudinally cut aortas coupled to a loading cell for force measurements with the Extroducer, scaffolded by iron tubing (fig. 2a). Initial penetration by the sharp tip is achieved at 11 gram. Full force to penetrate the vessel wall with the intrusion depth limiting collar is then applied, augmented by the scaffolding iron tubing. The vessel wall gives away at 45 gram. Computing a ratio between the forces needed for initial penetration and a then the collar, in essence, an overshooting of the system conveys that at least five times the force is needed. Testing maximum application of force until bending of the nitinol tubing occurred, showed that 20 mm outside the iron scaffold, the maximum force is 17 gram. (Data plot not shown). Therefore with regular plastic catheter scaffolding of the Extroducer system, it is impossible to overshoot the intrusion depth limiting collar past the vessel wall.

### Flow simulations and simulator testing

After detachment of the distal tip, the inner lumen would be open and could potentially cause hemorrhage in the target tissue. We performed flow calculations through different radii of a distal tip at a pressure of 200 mmHg depicted in the graph of figure 2b illustrating a circular Poiseuille flow, filled symbols, and with COMSOL Multiphysics calculations to integrate turbulent flow, open symbols, through a 2 mm lumen length. In the graph, flow rates of water, plasma and whole blood at 37°C and a physiological hematocrit of 45 (Y-axis) are plotted against the lumen radius (X-axis). Fluid mechanics states that the flow rate out of the lumen varies as the fourth power of the radius. Consequently, the flow rate quickly becomes small as the lumen radius is reduced. At a radius of 50 micrometer, the results of the two methods coincide but at higher radii; the open symbols show lower flow rates because turbulent flow is taken into account in calculations with COMSOL Multiphysics. In practical terms, this gives an auto-sealing effect up to 100 µm radius where flow is in the range of 100 µl per second at physiological blood pressures before introducing coagulation in the model at low and/or turbulent flow.

To further illustrate impact of turbulent flow, fig. 2c depicts a graph illustrating velocity fields of circular Poiseuille flows, filled symbols and COMSOL Multiphysics, open symbols, in an Extroducer device having a 2 mm long lumen, wherein the velocity fields of whole blood, at 37°C and a physiological hematocrit of 45, driven by a pressure of 200 mmHg (Y-axis) are plotted against different lumen radius (X-axis). Turbulence reduces the velocity in the central part of the velocity fields. For a radius of 50 micrometer, the result by COMSOL Multiphysics is identical to the circular Poiseuille flow, indicating full laminar flow for this geometry whereas the result by COMSOL Multiphysics is slightly reduced compared to the Poiseulle flow at a radius of 100 micrometer and heavily reduced at radii of 150 and 200 micrometer, respectively. When simulating water flow (lower viscosity than whole blood), with assistance of COMSOL multiphysics, through the detached Extroducer with an inner luminal radius of 100 µm (double that of our prototype), no laminar flow was observed with a driving pressure of 300 mm Hg (39.9 kPa).

The Extroducer concept is thus compromised of a depth limiting collar for adequate placing. Directly proximal to that is a hollow detachment zone and when detached no leakage through the interior lumen occurs for the smallest fabrications. For larger diameters the lumen is sealed by pushing a cylinder to the Extroducer tip prior to detachment. Integration was then tested in our simulator environment of the human vascular tree connected to rat carcasses. Navigations through the simulator system were all successful with full compatibility towards the clinical setting.

### Extroducer *in vivo* testing - small animals

Short term testing was performed in rat by creating arterial access from the medial tail artery and performing the Extroducer transvascular passage in either the subclavian or carotid artery testing two different stages; the transvascular passage and the deployment of a tip. No cases of intra-operative hemorrhage or intra-luminal thrombosis occurred. Thus, the vascular penetration procedure was uneventful and the vessel wall completely sealed around the Extroducer thereby preventing leakage of blood. When applying papaverin to resolve potential vasospasm due to the penetration procedure, no hemorrhage or other complications were observed over a 90 minutes period. The animals excluded as non-successful navigations were all excluded on the basis of navigation solely with the nitinol guide and plastic tubing prior to advancement of the penetrating prototype.

**Figure 3:**
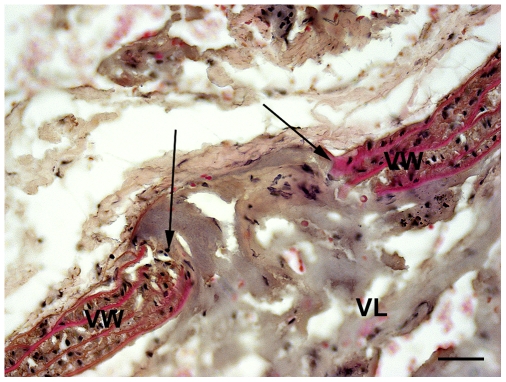
Histology of vessel perforation. Microphotograph showing the vessel perforation made by the Extroducer body with an outer diameter of 194 µm. The Extroducer makes a penetration that is on average 70 µm in diameter, indicated by arrows in this histological vessel sample stained by hematoxilin and eosin. VW indicates vessel wall and VL indicates vessel lumen, respectively. Scale bar: 20 µm.

Histological analyses of vessels (fig. 3) showed an average penetration diameter of 70 µm for the smallest dimension tested (outer diameter 193 µm±0.0127 µm).

The group with deposited Extroducer tips lacking intrusion depth limiting collars also showed absolute hemostasis during the primary intervention. Fourteen days post intervention, this group showed no signs of pain or discomfort. No signs of dissection of the vessels or impairment of blood-flow distal to intervention sites were observed and macroscopical analysis of the organ supplied by the vessel, showed no infarcts. The flow simulations indicating absence of blood-flow through the interior lumen was also tested *in vivo* by cannulating the detached transvascularly positioned tip of the prototypes with a nitinol mandrel. This was done to reassure that even when removing clotting inside the prototype it still prevented bleeding from inside the vessel to the extravascular space. Furthermore, no signs of delayed hemorrhage were detected. The Extroducer prototypes were found associated to the outside vessel wall or in the extra-vascular space adjacent to the penetration site, indicating that the deposited Extroducer tip is actively “pushed” out of the vessel wall by the pressure gradient when lacking a depth limiting collar.

### Extroducer *in vivo* testing - large animals

The Extroducer system tested in large animals was manufactured from a longer nitinol tube, 1700 mm *vis-à-vis* 300 mm that was used in the rat and with a smaller depth-limiting collar. We evaluated the prototypes together with clinical standard catheters and angiographic equipment in the rabbit. A plastic catheter around the Extroducer was used to avoid cutting the clinical microcatheter when advancing the Extroducer system, and for additional isolation during detachment.

**Figure 4:**
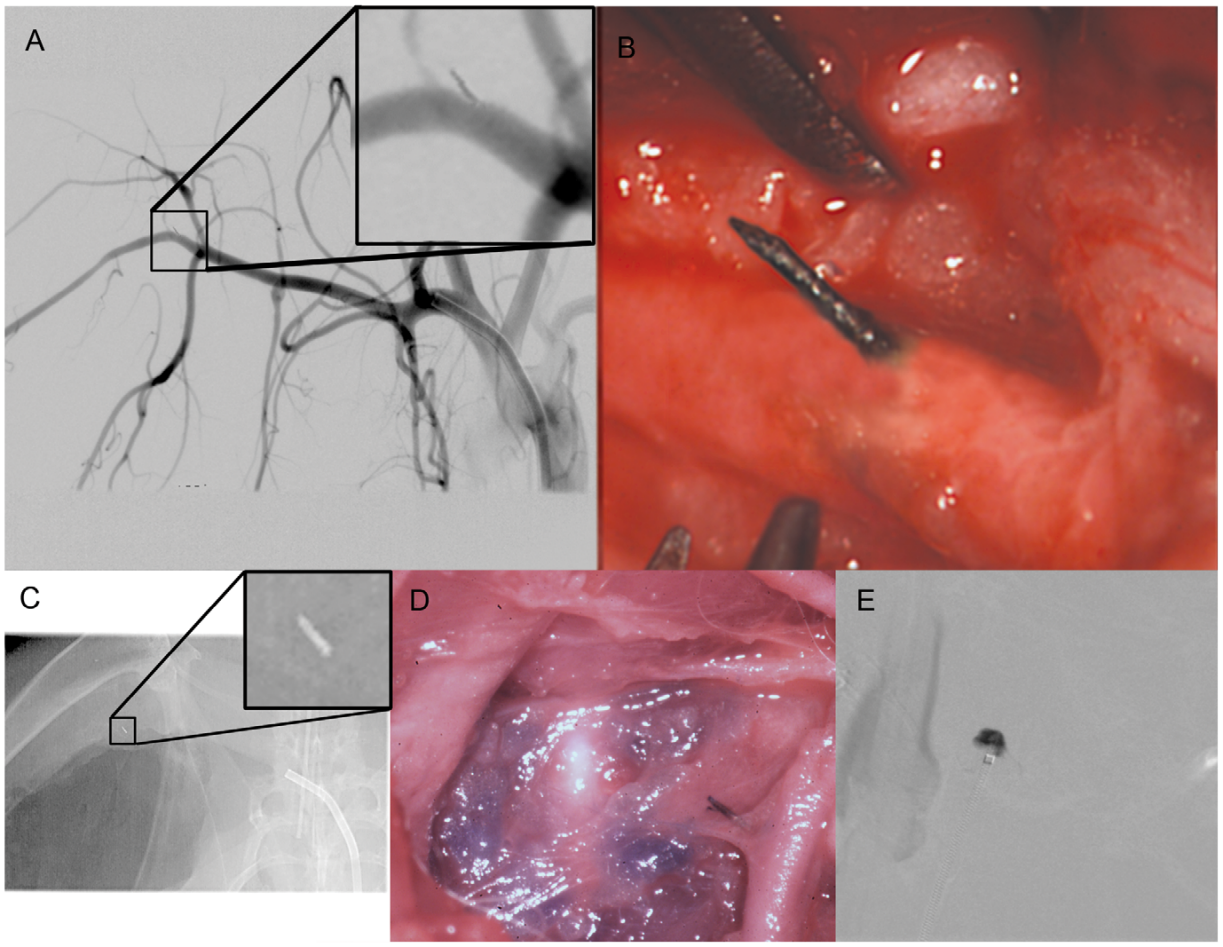
Radiological and microsurgical examples of the intervention. For full control over the procedure in the large animal trials, both a surgical microscope and high resolution angiographical series was used. In a. digital subtraction angiogram showing a detached Extroducer tip without hemorrhage, dissection or thromboembolic complications. In b. photograph showing the microsurgical view of the detached Extroducer tip. In c. x-ray image showing the detached Extroducer tip with guide catheter. In d. photograph from post-operative dissection showing the detached Extroducer tip with methylene blue injected in the surrounding tissue. In e. digital subtraction angiogram showing an extra vascular injection of 25 µl contrast agent through the Extroducer system.

The Extroducer prototypes were visible at high magnification fluoroscopy and thereby maneuvered into position. A slight amount of pressure was required on the plastic catheter to advance it to the desired vessel wall and thereafter the Extroducer was gently advanced out to the extra-vascular space (Fig. 4) (n = 40). No hemorrhages (0%) were observed by simultaneous direct observation through a surgical microscope, and no hemorrhages, thrombo-embolic complications or dissections were observed using high resolution angiographic series, during and after the intervention. No navigational problems were encountered with respect to Extroducer prototype integration with clinical catheters.

**Figure 5:**
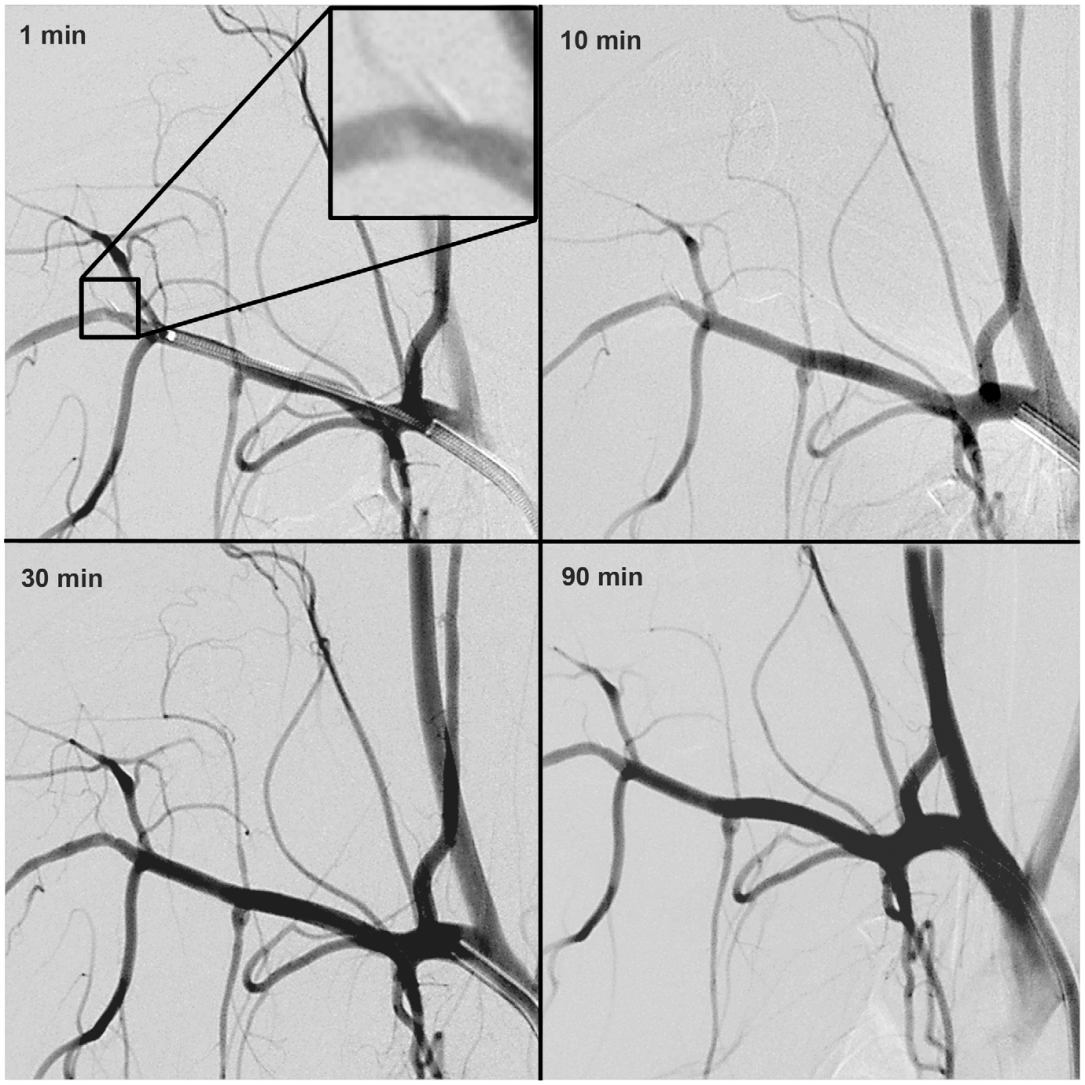
Follow up digital subtraction angiography. Digital subtraction angiography series in the subclavian artery at different time points after detachment of the Extroducer tip.

Application of papaverin did not cause hemorrhage, as expected based on the small animal testing. In high resolution angiographic series obtained up to 90 minutes after perforation, no effects at the perforation site or in the circulation distal to the penetration site could be observed (Fig. 5). However, when deliberately provoking bleeding by retracting the Extroducer prototypes without prior detachment, hemorrhages were observed.

Finally, the electrolysis detachment was tested in rabbit (n = 6). After navigation to the designated intervention site, methylene blue or contrast agent was deposited (Fig. 4), a tension of 8 V was applied and the distal end detached after on average five minutes (range three to nine minutes). This was also un-eventful without observation of hemorrhage both from around the body of the distal tip or through the inner lumen. The procedure was successfully performed both with simultaneous microscopic monitoring via surgical access, and with fluoroscopical/angiographical guidance solely.

## Discussion

Testing the different Extroducer prototypes in simulation, *ex vivo* and *in vivo* both in small and large animals have shown that the Extroducer design is a safe way of performing interventions in order to create a working channel to a target organ by endovascular technique. The novel design of the system in this study combines the minimally invasive properties of endovascular intervention and the creation of a working channel similar to an open surgical or percutaneous approach. One of many possible applications is optimal transplantation of insulin producing β-cells which hitherto has been performed by intraluminal injection in the portal vein[2], [16]. We hypothesize that our system could add the benefit of a more accurate placement of the cells while avoiding the surgical risks of open transplantation.

From the small animal tests we conclude that it is an intrinsic property of the vessel to prevent hemorrhage for Extroducer perforations. We further conclude that with today's limitations for endovascular tubing, the Extroducer principle works for vessels with diameters down to around 0.5 to 1 mm. However, we assume that this property applies to vessels of all sizes based on experiences from clinically used introducer systems.

The rationale for the intrusion depth limiting design is that, even in high magnification fluoroscopy, the resolution for optimal control is not sufficient. It is thus a safety precaution to avoid the risk for overshooting the system. The dimension is designed, and verified by the loading cell tests. Considering the forces measured, it is actually impossible to reach levels needed for overshooting the system because the nitinol in the body will bend without scaffolding from a steel pipe; in the clinical system it is only scaffolded by the SUBL tubing or a correspondently elastic catheter. The Extroducer detached distal part can easily be made long if the extra-vascular target zone is located far from the penetrated vessel.

For the smallest fabrications of the Extroducer, blood flow through the detached tip causing hemorrhages did not occur *in vivo*, as predicted by simulations. Furthermore, the risk of an inner lumen blood-flow is heavily exaggerated in our computer simulations since we assume a perfect flow entering the distal tip whereas *in vivo*, the flow will be heavily turbulent upon entering the detached tip because of the large angle between the blood vessel and the Extroducer.

Considering the tested dimension of the Extroducer system, it is possible to perform these interventions in all vascularised parts of the body with excellent accuracy. We decided not to miniaturize even further primarily due to current standards in manufacturing nitinol alloy tubing. A silicon based Extroducer could be constructed even smaller but that would probably bring about troubles when working with cells since exposing cell suspensions to large shear stress would cause necrosis. Furthermore, the Extroducer when placed inside a plastic tubing is small enough to fit in standard micro-catheters allowing navigation to desired areas with conventional techniques. The full scope of endovascular guiding and micro-catheters available today is thus usable with the current prototype size. On the other hand, the design of the system allows for easy up-scaling which makes vascular exit possible also in considerably larger luminal diameters. For larger Extroducer dimensions, where a leakage would occur through the inner lumen, a plug is advanced beyond the detachment zone, thereby preventing hemorrhage. In further developments of the system, the distal tip could be made in a biodegradable material.

Long term studies on vessel effects will be required for evaluation of possible stenosis forming at the area of the detached distal tip, thrombo-embolic complications or tracking of a possible displacement of the tip itself. However, clinical experience has shown that for example small nitinol wires or stents protruding into the vascular lumen do not cause such problems.

For cell transplantation purposes, the full range of the Extroducer system is the ability to perform accurate placing of engraftments and use smaller number of cells with minimally invasive procedures. If a cell-type has a proven property of homing only to a designated area, an intravenous/intra-arterial infusion could be preferable. However, to minimize non-specific spreading of transplanted cells and guarantee proper anatomical location, the Extroducer has clear advantages.

For the purpose of administrating substances to organs with difficult surgical access one can think of many applications ranging from cytostatics, contrast agents, growth factors, isotopes etc. The system could also be used for sampling procedures either for fluids with small diameter applications of the system, or tissue/cells with larger diameter applications combined with micro-forceps.

In conclusion, the novel design of the Extroducer prototypes has sustained all testing so far. We consider the Extroducer, in a potential clinical setting, a valuable tool for creating direct access to all organs of the body with minimally invasive endovascular technique for parenchymal access to perform stem cell transplantations, cytostatics or contrast agent injections or cytological sampling. The benefit should be greatest in organs that are difficult or high risk to access with other techniques, such as the pancreas, the central nervous system and the heart.

## Materials and Methods

### Ethic statement

All animal studies were conducted according to Karolinska Institutet guidelines of animal experiments on small rodents and rabbits. The studies were approved by the regional ethics committee for animal research at the Karolinska University Hospital (Stockholm, Sweden).

### Initial testing of concept

The first testing of the concept was performed by sharpening an ultra-thin super-elastic nitinol tube with outer diameter 0.193 mm±0.0127 mm, inner diameter 0.104±0.0127 mm (Tube NiTi SE 508, ground surface, Euroflex GmbH, Germany) and connecting this to a 50 µl micro syringe. This catheter was then used to perforate a rat tail artery from the outside and then immediately go through out via the opposing vessel wall.

### Penetration depth limiting design

Design simulations were performed in COMSOL Multiphysics software (Comsol AB, Sweden). Outer penetration depth limiting designs were constructed with copper treading and tested according to fig. 1. These stops were then tested in a system, scaffolded by iron tubing (ID 270 µm), on rat aorta *ex vivo*, cut longitudinally and hanging in free air between two connecting points on a cork plate (n = 10). Care was taken to perform this immediately after dissection of the vessels so they would not dry. The cork plate was connected to a loading cell 1004-300 (AB Svenska våg, Sweden) which in turn was connected to a weight instrument LD5208 (AB Svenska våg, Sweden) giving real time output to a computer recoding the force applied in the perpendicular direction of the inside of the vessel. Hereby forces could be measured for vascular penetration with the grinded nitinol tip and different depth limiting collars. Ratio calculations could be performed between penetration by the distal portion and different depth limiting designs.

### Hollow detachment zone

To construct the hollow detachment zone and subsequent simulation testing we used a 1700 mm long nitinol tube that was cleaned with alcohol. For detachment zones, we applied parylene through gas polymerization in vacuum in a 3 µm thickness (ParaTech Coating AB, Sweden). Directly proximal from the depth limiting collar a small insulation defect was cut through the parylene. For *in vitro* testing, prototypes were submerged in isotonic solution with 8 V tension through an electrode (cathode) and prototypes (anode) (n = 20). A tension of 8 V was applied by an external electrical source resulting in a circumferential dissolution of the catheter where the cut in the parylene was made. The distal penetration end was then separated from the proximal access portion of nitinol tube. The system used *in vivo* was constructed in a similar manner but the cathode was a needle placed in the hind limb of the rabbit.

### Stem cell survival in small dimensions

Effects on stem cells were tested as previously described [11]. Briefly, cells were passed through a 1700 mm Extroducer prototype and live and dead cell ratios were calculated before and after passage through the addition of Trypan blue to the cell suspension (Invitrogen, United Kingdom) and counting four square millimeters of living and dead cells in a Bürker chamber.

### Flow simulations in small dimensions

Blood flow from inside the vascular tree out to the extra-vascular space via the lumen of the prototypes was assessed with two types of simulations. We performed calculations of a circular Poiseuille flow[18] and another simulation by COMSOL Multiphysics which takes into account turbulent flow, wherein we included water, plasma and whole blood with a hematocrit of 45. These fluids were assumed to have a viscosity of 6.17E-04 Pa·s, 1.5E-03 Pa·s and 3.2E-03 Pa·s respectively[18], all at 37°C. We also performed velocity fields calculations of whole blood, driven by a pressure of 200 mmHg for different lumen radius in both COMSOL Multiphysics and with perfect circular Poiseuille flow.

### Extroducer simulations and *ex vivo* experiments

A simulation environment were constructed to test 1700 mm long superelastic nitinol alloy tubes with diameters as described earlier inside standard clinical guide- and micro-catheter systems. A simulator of the human femoral, illica, aorta, truncus coelicus and hepatic artery was constructed by connecting plastic tubing of decreasing size and mounting them on a plate. The distal end corresponding to the hepatic artery was then introduced in rat carcasses through the distal end of the abdominal aorta for *ex vivo* testing. Standard 6 F clinical introducer, guiding catheter; Envoy (Cordis, USA) and micro-catheter; Prowler Plus (Cordis, USA) were used to navigate within the simulator. Herein we tested the maneuverability of the system and if enough driving pressure could be applied to penetrate vessel walls. This was done on two rat aortas with approximately 30 penetrations each.

### Extroducer *in vivo* testing, small animals

A total number of 19 male Sprague-Dawley rats (BW 240–350 g; B&K Universal AB, Sweden) were included in the study. Two groups of small animals were used. One group with five animals that only tested hemorrhage in or around the interventional area and a second group with seven animals with survival time 14 days where we simulated detachment of the Extroducer tip by leaving the distal portion through the vascular wall after penetration to analyze relative long term effects.

Six non-successful navigation animals of the subclavian or left common carotid artery were excluded from their groups and euthanized via decapitation under the same anesthesia session.

Surgical anesthesia was performed by an intramuscular (im) injection of 0.2 ml Hypnorm-Dormikum (1∶1∶2; Hypnorm (fentanyl citrate 0.315 mg/ml, fluanisone 10 mg/ml, Janssen Pharmaceutical, Belgium): Dormikum (midazolam 1 mg/ml, Roche AB, Sweden)). Prior to skin incision, 0.1 ml Marcaine (5 mg/ml, AstraZeneca, Sweden) was injected subcutaneously in the area of operation. Animals were anesthetized with 0.1 ml intra-muscular Hypnorm before decapitation.

In the rat we tested a 300 mm long nitinol prototype that was introduced through the rat tail artery within a plastic PTFE-190 Sub-Lite wall tubing (SUBL) with outer diameter of 482±0.025 µm and inner diameter of 330±25 µm (AgnTho's, Sweden). The plastic tubing was used to minimize damage to the vascular tree by the sharp penetrating tip.

All small animal surgery was performed with a M651 operating microscope (Leica AB, Sweden) coupled to a CCD camera (Sony AB, Sweden).

Introduction of catheters were performed via the medial tail artery. A small longitudinal incision was cut on the ventral part of the tail through the skin and the fascia overlying the artery. A ligature was used to secure the PTFE-190 tube containing nitinol leader and then the catheter system was blindly navigated up through the aorta.

For observation and usages of the Extroducer prototypes, open surgical preparation of either the common carotid artery via a small mid-line incision medially on the neck, or the subclavian artery via an axillary exploration were performed. To maximize surgical access and navigational success-rates both the major and the minor pectoral muscles were cut.

After navigation to the common carotid artery or the subclavian artery with the blunted nitinol tube inside the PTFE-190 tube, the nitinol was exchanged for an Extroducer prototype thereby protecting vessels from damage during navigation. After reaching tip to tip, the Extroducer prototype was gently advanced through the arterial wall (taking advantage of the vessels non-linear anatomy) (n = 20). For all prototypes, tests to exclude vasospasm as a potential hemostatic cause, were performed by soaking the perforated vessel with papaverin (Recip AB, Sweden) and observed for 90 minutes. Mechanical manipulation of the detached Extroducer was also performed to provoke possible hemorrhage.

In the second group, the same operational techniques were used but instead of the 300 mm long prototype a 2.5 mm long prototype was advanced through the vessel wall using a nitinol pusher (n = 7). The prototypes, deployed through the vascular wall were then cannulated with a nitiol wire (OD 90 µm, ground surface, Euroflex GmbH, Germany) to remove formed cloths and detect possible hemorrhage. Without outer hemostatic control and after application of papaverin these animals were sutured both for re-attaching the pectoral muscles and the skin and allowed to recover in home cages. 14 days later they were re-explored and euthanized.

### Extroducer *in vivo* testing, large animals

Two groups of rabbits were used in this study. In the first group four animals were used for transvascular Extroducer perforations were performed with fluoroscopical, angiographical and simultaneous open microsurgical monitoring with several interventions per animal (n = 40). In the second group four other animals were subject to Extroducer perforations performed with fluoroscopical and angiographical monitoring only, followed by methylene blue- and contrast agent injection through the nitinol tube at a flow rate of 2 to 5 microliters per second. The procedures were finished by detaching the distal Extroducer portion by electrolysis and removing the catheter system (n = 6). Post-euthanasia dissections were performed. Surgical anesthesia was induced by subcutaneous injection of 0.5 ml/kg Hypnorm (fentanyl citrate 0.315 mg/ml, fluanisone 10 mg/ml, Janssen Pharmaceutical, Belgium) combined with 5 mg diazepam. An intravenous line was established in the ear veins bilaterally. A bolus dose of Propofol was administered and thereafter, the rabbit was intubated with a size 3 pediatric tube and connected to a Siemens 900 servo ventilator (Siemens Healthcare, United Kingdom). The animal was infused with propofol according to standard rabbit doses, *i.e.* 22 mg/ml/h. In addition, 0.1 ml of Hypnorm was injected intravenously every 30 minutes. For euthanizing the rabbits, intra-venous Pentobarbital overdoses were used.

All large animal angiography and surgery was performed with a Philips XD20 angiographical equipment (Philips medical system, Netherlands) and a OPMI6-DF operating microscope (Carl Zeiss AB, Sweden) connected to a CCD camera. Visipaque 270 contrast agent (GE healthcare, USA), was used in all angiography applications.

The femoral artery of the anesthetized rabbit was exposed surgically and a 5 French Introducer was inserted in the artery (Terumo, USA). Under fluoroscopic and angiographic control, a 5 French Envoy guiding catheter (Cordis Corporation, USA) was navigated to different parts of the vasculature of the rabbit. A Prowler Plus microcatheter (Cordis Corporation, USA) or Renegade micro-catheter (Boston Scientific, USA) was inserted within the Envoy guiding catheter and together with a Transend Platinum Tip guidewire (Boston Scientific, USA) navigated under angiographic control to the microvasculature (0.5–1 mm in lumen diameter) in different parts, of the rabbit. After having reached the desired target location, the guidewire was withdrawn from the Prowler Plus microcatheter and the Extroducer within a PTFE-190 Sub-Lite wall was inserted and deployed through the arterial wall. This was performed both under direct visual control with surgical microscope and under high magnification fluoroscopic control with subsequent digital subtraction series. Injections with methylene blue and contrast agent were performed to additionally prove the concept of substance administration to the extra vascular space. Perforations were followed up to 90 minutes by high resolution digital subtraction angiographical series.

### Tissue preparation and Histochemistry

Vessels were clamped, cut and fixated for 72 hours in 4% buffered paraformaldehyde at 4°C. They were placed in 15% sucrose for 24 hours at 4°C following the fixation procedure. The vessels was then mounted on a holder and frozen in a Leica cryostat (CM 3000, Leica Instruments GmbH, Germany). They were covered with mounting medium and cut in 10 µm sections and thaw mounted onto Super Frost/Plus object glasses (Menzel-Gläzer, Germany). The sections were stained with Hematoxylin and Eosin[19]. Slides were then analyzed with a Leica DM 4000 B microscope and photographed with a coupled Leica DFC 320 CCD camera. Quantification of perforations in vascular wall was measured using ImageJ (Open-source software, NIH, USA).

## References

[1] Bale R, Widmann G (2007). Navigated CT-guided interventions.. Minim Invasive Ther Allied Technol.

[2] Villiger P, Ryan EA, Owen R, O'Kelly K, Oberholzer J (2005). Prevention of bleeding after islet transplantation: lessons learned from a multivariate analysis of 132 cases at a single institution.. Am J Transplant.

[3] Bliss T, Guzman R, Daadi M, Steinberg GK (2007). Cell transplantation therapy for stroke.. Stroke.

[4] Guzman R, Choi R, Gera A, De Los Angeles A, Andres RH (2008). Intravascular cell replacement therapy for stroke.. Neurosurg Focus.

[5] Wu XH, Liu CP, Xu KF, Mao XD, Zhu J (2007). Reversal of hyperglycemia in diabetic rats by portal vein transplantation of islet-like cells generated from bone marrow mesenchymal stem cells.. World J Gastroenterol.

[6] Hampton T (2007). Stem cells ease Parkinson symptoms in monkeys.. Jama.

[7] Schachinger V, Assmus B, Britten MB, Honold J, Lehmann R (2004). Transplantation of progenitor cells and regeneration enhancement in acute myocardial infarction: final one-year results of the TOPCARE-AMI Trial.. J Am Coll Cardiol.

[8] Mahmood A, Lu D, Qu C, Goussev A, Chopp M (2006). Long-term recovery after bone marrow stromal cell treatment of traumatic brain injury in rats.. J Neurosurg.

[9] Shen LH, Li Y, Chen J, Cui Y, Zhang C (2007). One-year follow-up after bone marrow stromal cell treatment in middle-aged female rats with stroke.. Stroke.

[10] Jin K, Sun Y, Xie L, Mao XO, Childs J (2005). Comparison of ischemia-directed migration of neural precursor cells after intrastriatal, intraventricular, or intravenous transplantation in the rat.. Neurobiol Dis.

[11] Lundberg J, Le Blanc K, Soderman M, Andersson T, Holmin S (2009). Endovascular transplantation of stem cells to the injured rat CNS.. Neuroradiology.

[12] Kelly S, Bliss TM, Shah AK, Sun GH, Ma M (2004). Transplanted human fetal neural stem cells survive, migrate, and differentiate in ischemic rat cerebral cortex.. Proc Natl Acad Sci U S A.

[13] Wennersten A, Meier X, Holmin S, Wahlberg L, Mathiesen T (2004). Proliferation, migration, and differentiation of human neural stem/progenitor cells after transplantation into a rat model of traumatic brain injury.. J Neurosurg.

[14] Thompson CA, Nasseri BA, Makower J, Houser S, McGarry M (2003). Percutaneous transvenous cellular cardiomyoplasty. A novel nonsurgical approach for myocardial cell transplantation.. J Am Coll Cardiol.

[15] Siminiak T, Fiszer D, Jerzykowska O, Grygielska B, Rozwadowska N (2005). Percutaneous trans-coronary-venous transplantation of autologous skeletal myoblasts in the treatment of post-infarction myocardial contractility impairment: the POZNAN trial.. Eur Heart J.

[16] Merani S, Toso C, Emamaullee J, Shapiro AM (2008). Optimal implantation site for pancreatic islet transplantation.. Br J Surg.

[17] Seldinger SI (1953). Catheter replacement of the needle in percutaneous arteriography; a new technique.. Acta radiol.

[18] Fay JA (1994). Introduction to Fluid Mechanics..

[19] John D, Bancroft AS (1996). Theory and Practice of Histological Techniques..

